# Exopolysaccharide II Is Relevant for the Survival of *Sinorhizobium meliloti* under Water Deficiency and Salinity Stress

**DOI:** 10.3390/molecules25214876

**Published:** 2020-10-22

**Authors:** Emiliano Primo, Pablo Bogino, Sacha Cossovich, Emiliano Foresto, Fiorela Nievas, Walter Giordano

**Affiliations:** Instituto de Biotecnología Ambiental y Salud (INBIAS), CONICET, Departamento de Biología Molecular, Facultad de Ciencias Exactas, Físico-Químicas y Naturales, Universidad Nacional de Río Cuarto (UNRC), Río Cuarto X5804BYA, Córdoba, Argentina; eprimo@exa.unrc.edu.ar (E.P.); pbogino@exa.unrc.edu.ar (P.B.); scossovich@exa.unrc.edu.ar (S.C.); eforesto@ayv.unrc.edu.ar (E.F.); fnievas@exa.unrc.edu.ar (F.N.)

**Keywords:** *S. meliloti*, exopolysaccharides, water deficiency stress, saline stress, biofilm, autoaggregation

## Abstract

*Sinorhizobium meliloti* is a soil bacterium of great agricultural importance because of its ability to fix atmospheric nitrogen in symbiotic association with alfalfa (*Medicago sativa*) roots. We looked into the involvement of exopolysaccharides (EPS) in its survival when exposed to different environmental stressors, as well as in bacteria–bacteria and bacteria–substrate interactions. The strains used were wild-type Rm8530 and two strains that are defective in the biosynthesis of EPS II: wild-type Rm1021, which has a non-functional *expR* locus, and mutant Rm8530 *expA*. Under stress by water deficiency, Rm8530 remained viable and increased in number, whereas Rm1021 and Rm8530 *expA* did not. These differences could be due to Rm8530′s ability to produce EPS II. Survival experiments under saline stress showed that viability was reduced for Rm1021 but not for Rm8530 or Rm8530 *expA*, which suggests the existence of some regulating mechanism dependent on a functional *expR* that is absent in Rm1021. The results of salinity-induced stress assays regarding biofilm-forming capacity (BFC) and autoaggregation indicated the protective role of EPS II. As a whole, our observations demonstrate that EPS play major roles in rhizobacterial survival.

## 1. Introduction

Microbial EPS are defined as polysaccharides produced by microorganisms and secreted out of the cell, which have a significant role in the protection of the cell and in cell-to-cell and cell-to-surface interactions. They are mainly made up of carbohydrates (a wide range of sugar residues) and some non-carbohydrate constituents, such as phosphate, acetate, pyruvate, and succinate [1,2]. Nevertheless, the composition, function, and physical properties that determine their primary conformation vary from one bacterial species to another.

*Sinorhizobium meliloti* is an alpha-proteobacterial model studied not only because of its nitrogen-fixing symbiosis with certain legumes, but also due to its close relationship with pathogenic species such as *Brucella* and *Candidatus* Liberibacter, which infect animals and plants, respectively. It synthesizes two types of extracellular polysaccharides, succinoglycan (EPS I) and galactoglucan (EPS II), whose polymerization results in the formation of two major fractions: one of low molecular weight (LMW), and another of high molecular weight (HMW). The LMW fraction, an active biological form of EPS, is essential for the successful infection of leguminous plants that form indeterminate-type nodules [3]. Under standard laboratory culture conditions, EPS I is the only EPS synthesized by the two reference strains, Rm1021 and Rm2011 [3,4]. In both strains, EPS II synthesis was observed under phosphate-limiting conditions [5], or under any of the three genetic conditions required to activate its production: (i) restoration of the functionality of regulator gene *expR* [4], (ii) a mutation in the gene regulator *mucR* [5], or (iii) the presence of extra copies of genes in the cluster *exp* [5]. The quorum sensing (QS) regulator coded by *expR* [6,7], moreover, controls the expression of several genes in *S. meliloti* [8]. The ExpR/Sin QS system, for instance, is involved in the regulation of genes responsible for EPS I biosynthesis as well [9].

In natural environments, different signals govern events that stimulate bacteria to form architecturally complex and organized communities called biofilms [10]. These multicellular conglomerates are embedded in a matrix produced by the bacteria themselves and can often be found adhered to both biotic and inert surfaces. In fact, the extracellular matrix itself consists of EPS, proteins, lipids, and extracellular DNA. According to levels of specialization, intercellular communication mediated by QS, and coordinated behavior, a bacterial biofilm may be considered an organized and dynamic social structure [11]. Biofilm formation may be preceded by another phenomenon, autoaggregation, whose phenotype is also dependent on EPS synthesis [12] and which offers competitive advantages for survival as well, especially under the harsh conditions commonly found in soil environments [13]. Autoaggregation is a result of adhesive interactions between bacteria, which take place thanks to the fimbrial and afimbrial adhesins in bacterial cell walls [14]. This sessile lifestyle offers protection against harmful environmental agents (e.g., antibiotics, protozoa, desiccation, UV radiation, and toxic substances), and greater nutrient availability, as well as the possibility of metabolic cooperation with other species and acquisition of genetic material [15]. EPS perform crucial roles for microbial cells living in communities. Aggregative EPS act as molecular glue, allowing the bacterial cells to adhere to each other as well as to surfaces. One of the many benefits provided by this EPS component is protection against biotic and abiotic stress, and thus greater resistance. This means that EPS are significantly involved in the way that the cells in the biofilm interact with the surrounding environment [16,17], and they have been confirmed to intervene in rhizobial biofilm formation [18,19].

In terms of rhizobial bacteria, specifically, we have previously described the importance of rhizobial cell surface components such as EPS, in combination with bacterial functional signals, for the processes of autoaggregation [20] and biofilm formation [14]. A positive correlation between biofilm-forming capacity (BFC) and autoaggregation was observed in native *S. meliloti* strains, which demonstrates that the phenotypes of both processes depend on the same physical adhesive forces [21]. A mutation in LPS in the presence or absence of EPS II, moreover, was found to lead to important changes in cell–cell and cell–surface interactions, as well as in the symbiosis with the host plant [22]. We have also shown that EPS are necessary for the adhesive interaction between the bacterial species and the alfalfa plant but may not necessarily improve symbiosis [23]. Other authors have pointed out that EPS may help rhizobia to adapt to stressful environmental conditions such as desiccation, and that they could promote the formation of mixed biofilms serving as consortia to improve nodule development and functioning [24]. When studying bacterial communities isolated from rhizospheric alfalfa soils, we had already observed that a decrease in water in soil led to the establishment of a community consisting of members better able to grow under desiccation stress [25].

On the basis of this background, the present work aimed to contribute to the existing knowledge about the importance of EPS in bacterial survival. Strains with differing abilities to synthesize EPS I and EPS II [4,20] were used; namely, two laboratory reference strains (*S. meliloti* Rm1021 and Rm8530, both derived from *S. meliloti* SU47) and a mutant (*S. meliloti* Rm8530 *expA*). The role of EPS in these strains was assessed in connection with bacterial viability, BFC, and autoaggregation under stress conditions of desiccation and salinity.

## 2. Results

### 2.1. EPS Production in S. meliloti

**Figure 1:**
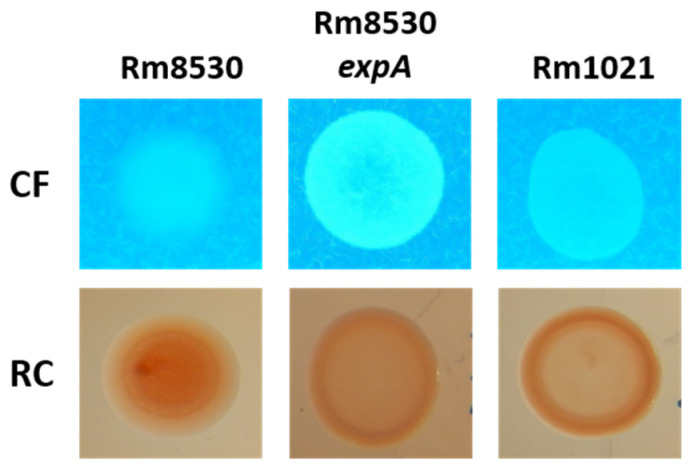
Comparisons between colony phenotypes in *S. meliloti* strains. Colony appearance for Rm8530, Rm8530 *expA* and Rm1021 grown in LB with 200 μg/mL CF white, and MGM agar supplemented with 125 µg/mL CR, into which inoculum drops were deposited. Plates were imaged after 48 h of growth at 30 °C.

Rm8530 has a mucoid phenotype dependent on EPS II synthesis, in contrast to the non-mucoid phenotype formed by Rm1021 and Rm8530 *expA*, two strains that do not produce EPS II due to insertions or mutation in different *exp* genes, respectively. Calcofluor (CF) UV-fluorescence and Congo Red (CR) on agar plates were used to detect EPS and distinguish between EPS I- and EPS II-producing bacteria (Figure 1). CF binds more specifically to ß(1-4) and ß(1-3) glycosidic bonds such as those in the EPS I synthesized by *S. meliloti* [26], whereas CR is less specific and binds to neutral or basic polysaccharides and some proteins [27]. After 48 h of growth, the colonies formed on the CF plates by all the strains assayed were fluorescent under UV light, although at different intensities, likely due to differences in the amount of EPS I that each strain produces [28]. On the CR plates, Rm8530 showed a mucoid phenotype and intense red colonies related to strong EPS production, but Rm1021 and Rm8530 *expA* were lightly colored in the center and surrounded by a red halo, which is indicative of a lower production (Figure 1).

### 2.2. Test of Viability in Sand: Water and Saline Stress

#### 2.2.1. Water Stress (Desiccation Assay)

**Figure 2:**
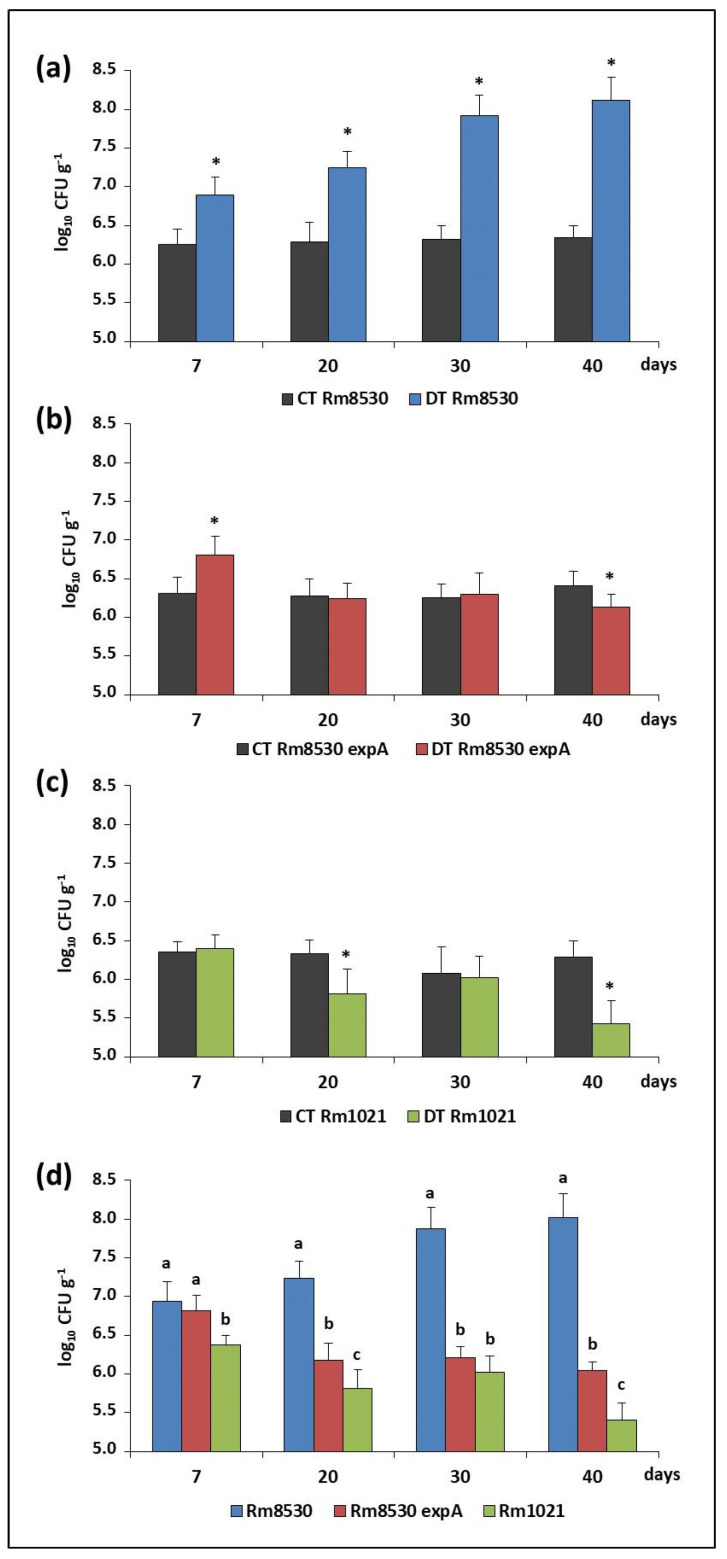
Survival of *S. meliloti* under water stress conditions. Bars show the average of viable cell counts (log_10_ CFU g^−1^) over time (7, 20, 30, and 40 days) using sterile sand as a matrix. Figures (**a**–**c**) show cell viability for Rm8530, Rm8530 *expA,* and Rm1021, respectively, under control (CT) and desiccation (DT) conditions. Figure (**d**) shows cell viability for each strain under desiccation over time. The error bars correspond to the standard deviations of five independent trials with three replicates each. According to Fisher’s LSD test (*p* ≤ 0.05), statistically significant differences between conditions (CT vs. DT; Figure **a**–**c**) for each point in time are indicated with asterisks, whereas statistically significant differences between strains are indicated with different letters (Figure **d**).

The wild-type reference and the mutant *S. meliloti* strains were subjected to water stress conditions (desiccation) and their viability was determined by counting the number of colony forming units per gram of sand (CFU g^−1^) at 7, 20, 30, and 40 days. At the beginning of the trial (day 0), the inoculum was 2 × 10^6^ CFU g^−1^ for all the strains evaluated. Under conditions of no desiccation (control tubes), there were no statistical changes across time in this initial bacterial inoculum for any of the strains tested. Under desiccation, CFU values for Rm8530 increased one-fold at the beginning of the trial (7 days) and two-fold at the end (40 days), which demonstrates that this strain was able to keep its viability. By contrast, relative CFU did not increase and was even reduced across time under the same conditions in the case of Rm8530 *expA* and Rm1021, both of which are incapable of producing EPS II. Figure 2 shows that strain Rm8530 was better able to survive under water stress conditions than non-EPS II-producing Rm5380 *expA* and Rm1021. Interestingly, the number of Rm8530 cells also increased throughout the assay, while the viability of the other two strains was reduced. These results suggest that the ability to produce EPS II gives Rm8530 an advantage over the other strains in terms of surviving in harsh environments.

#### 2.2.2. Saline Stress

**Figure 3:**
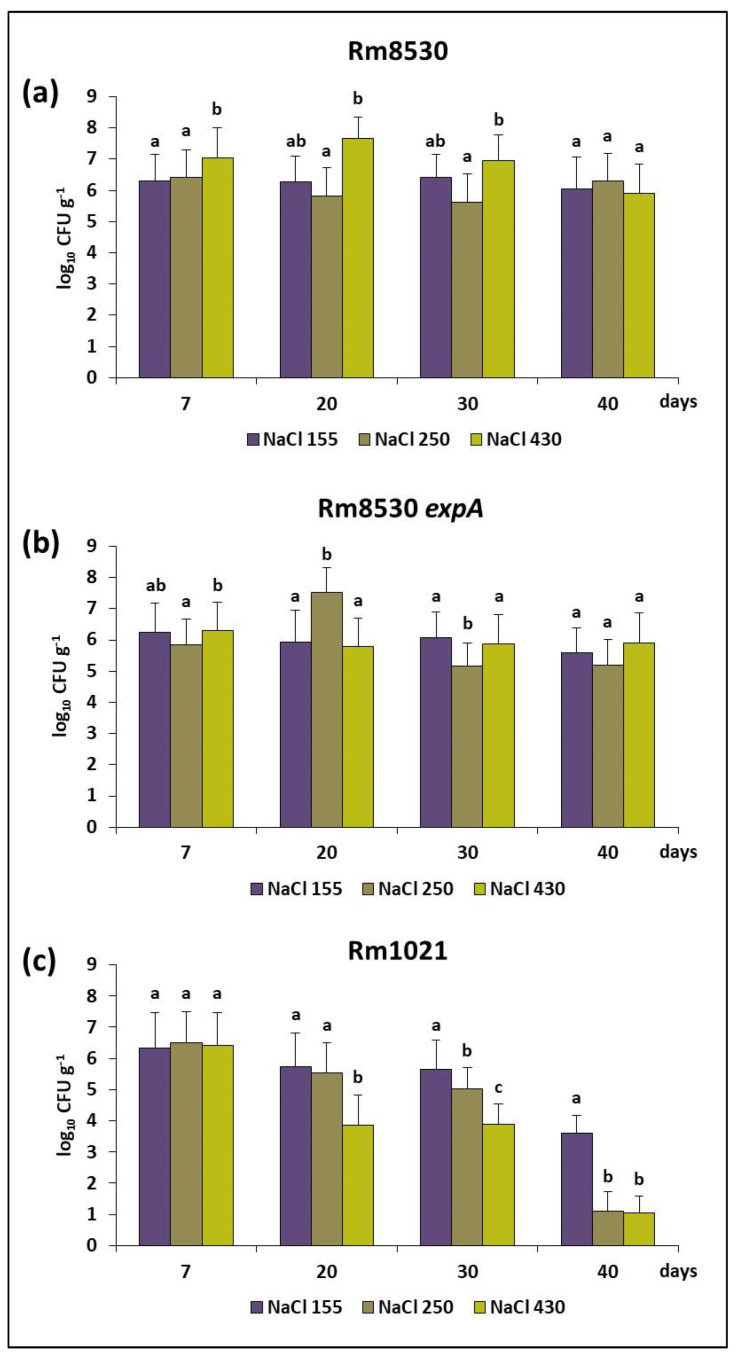
Survival of *S. meliloti* under saline stress conditions. Bars show the average of viable cell counts (log_10_ CFU g^−1^) over time (7, 20, 30, and 40 days) using sterile sand as a matrix, saturated with 155 mM, 250 mM or 450 mM NaCl solutions. Figure (**a**–**c**) show cell viability for Rm8530, Rm8530 *expA* and Rm1021, respectively. The error bars correspond to the standard deviations of five independent trials with three replicates each. According to Fisher’s LSD test (*p* ≤ 0.05), different letters indicate statistically significant differences between the three conditions for each point in time.

Different concentrations of NaCl (155 mM, 260 mM, and 430 mM), were used to assess the viability of the wild-type and mutant strains when exposed to saline stress. Rm8530 and Rm8530 *expA* maintained their viability over time under all saline stress conditions (Figure 3). On the other hand, the conditions created by the two higher concentrations of NaCl significantly reduced the survival of Rm1021. In this case, then, a connection between survival rate under saline stress and EPS II production cannot be made since both EPS II-producing Rm8530 and EPS II-defective Rm8530 *expA* maintained viability under saline stress. However, these two strains appear to share an important mechanism that regulates survival under salinity, which should be dependent on a functional *expR* which is not present in Rm1021.

### 2.3. Growth and Colony Phenotype of S. meliloti Strains

The use of NaCl as a diffusible solute to reduce water activity, and thus water potential, has been shown to trigger different bacterial mechanisms of survival under such conditions [29,30,31].

We analyzed the growth of the *S. meliloti* strains in solid LB media 0.25X (water potential Ψ −0.15 MPa) supplemented with different amounts of NaCl to decrease water potential (Ψ: −0.5 MPa, −1.0 MPa, −1.5 MPa, and −2.5 MPa).

**Figure 4:**
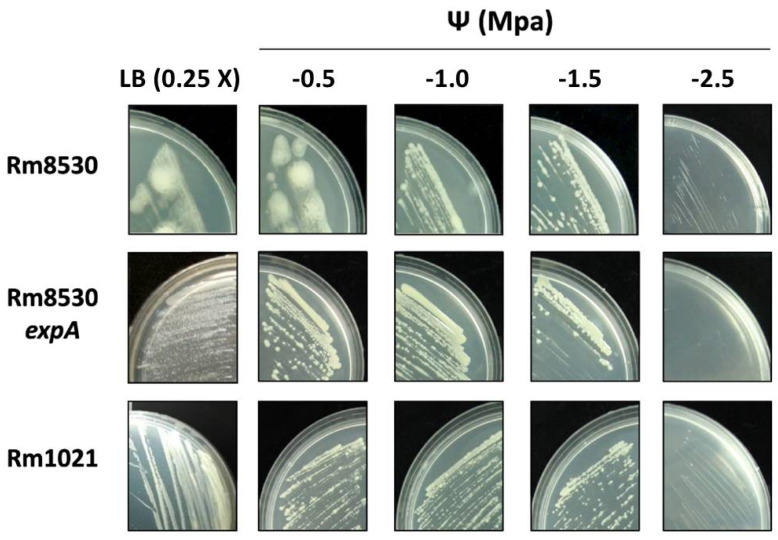
Growth and colony morphology under saline stress conditions. The figure shows the behavior of different *S. meliloti* strains grown on LB (0.25X) plates added with different amounts of NaCl to reduce water potential (Ψ). Growth and phenotype of colony morphology (mucoid or dry) can be seen.

Table 1 presents semi-quantitative results regarding growth levels in solid medium and colony phenotype under the conditions tested. In general, a severe reduction in water potential in the solid medium (−1.5 and −2.5 MPa) negatively affected the growth of all the strains. At −2.5 MPa, Rm8530 *expA* had no detectable growth after 5 days of incubation, while growth for Rm8530 and Rm1021 was slightly discernible (Table 1, Figure 4). As a whole, reference wild type strains Rm8530 and Rm1021 were more tolerant to the decreased water potential induced by NaCl.

In terms of colony phenotypes, those corresponding to Rm1021 and Rm8530 *expA* were dry and opaque, with defined edges under all the conditions analyzed (Table 1, Figure 4). The characteristic mucoid and bright phenotype with undefined edges of the Rm8530 colony became dry when exposed to water potential reductions equal to or lower than −1.0 MPa (Figure 4). This could be related to a negative inhibition of EPS II-synthesis regulation. Similar results were obtained for *R. meliloti* EFB1, in which a significant reduction in the amount of secreted EPS resulted in mucoid loss in its colonies [32].

### 2.4. Biofilm-Forming Capacity (BFC)

**Figure 5:**
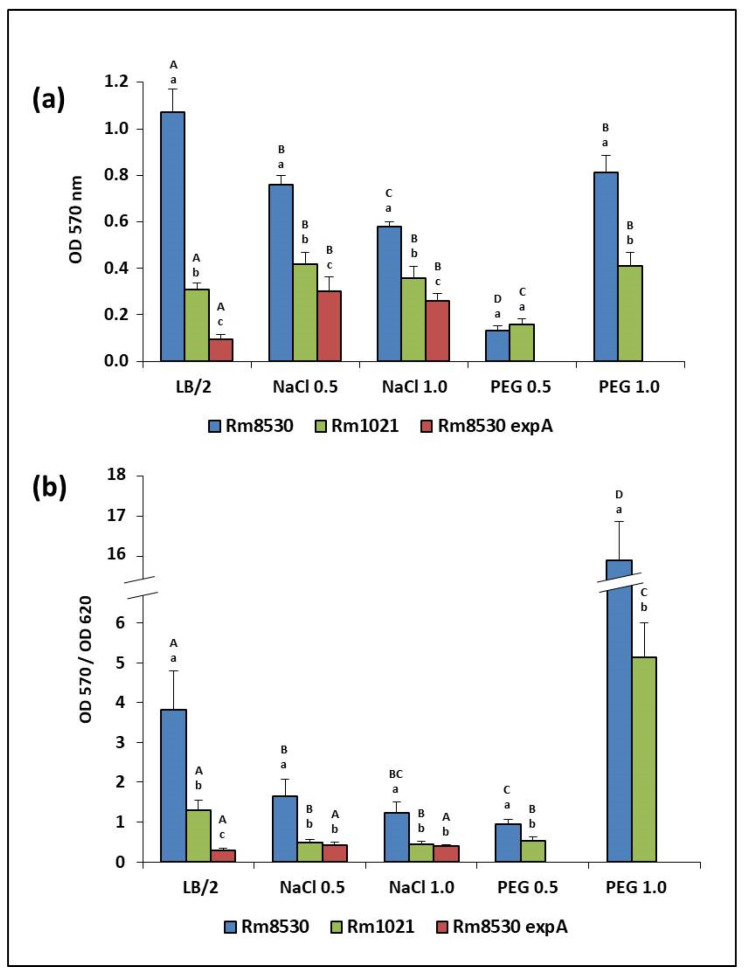
BFC of different *S. meliloti* strains under saline and matric stresses. (**a**) Biofilm development on polystyrene surfaces shown as an average of OD measurements at 570 nm. (**b**) Relation between biofilm and growth shown as the ratio between OD at 570 and 620 nm, respectively. According to Fisher’s LSD test (*p* < 0.05), small letters indicate statistically significant differences between strains under the same condition; capital letters indicate statistically significant differences for the same strain under different conditions.

BFC was assessed both by quantifying bacterial adhesion to a polystyrene support (OD570) and estimating the biofilm/growth (B/G) ratio (OD570/OD620). The behavior of *S. meliloti* varied between the wild-type and mutant strains used here, as well as under different stress conditions. Although Rm8530 was generally better at biofilm forming than the other two strains under all conditions evaluated (Figure 5a,b), this ability decreased under saline stress, between 20% (under NaCl −0.5 MPa) and 40% (under NaCl −1.0 MPa) with respect to the medium without added solute (Figure 5a). This effect was particularly more marked for the conditions created by PEG −0.5 MPa, under which Rm8530′s BFC decreased almost 90% in comparison with non-stressful conditions. By contrast, salinity created by NaCl increased the BFC of the strains unable to synthesize EPS II (Rm1021 and Rm8530 *expA*) by ~50% with respect to the non-stressful conditions (Figure 5a). When the stress was caused instead by the non-diffusible solute (PEG), the behavior of Rm1021 varied. Its BFC was slightly lower under PEG −0.5 MPa than under non-stressful conditions, but similar to the values recorded under saline stress when PEG −1.0 MPa was used. For its part, Rm8530 *expA* was unable to form biofilm under PEG conditions in our experimental conditions.

In spite of these observations about BFC, the B/G ratio can lead to more accurate conclusions about biofilm development since it is a normalized parameter for biofilm formation when the method of determination is based on CV measurements. The B/G ratio can be interpreted as a parameter of bacterial lifestyle under certain conditions. In the absence of stress, this ratio was higher for the strain producing EPS II (Rm8530), than for non-EPS II producers Rm1021 and Rm8530 *expA*. Even though growth parameters were good for all three, the increased BFC of Rm8530 determined its higher B/G ratio (Figure 5b). Under saline stress, the reduction in growth and biofilm formation did not produce high B/G ratios, but Rm8530 still had higher ratios than the other strains (Figure 5b). In the case of water stress induced by PEG (defined as matric stress), the effect varied according to the concentrations of the compound and the reductions they caused in water potential. For a −0.5 MPa decrease, BFC and growth were lower for all strains and therefore so was the B/G ratio. Under −1.0 MPa PEG, Rm8530 was capable of directing most of its biomass to the formation of biofilm. For this strain, stronger matric stress led to a B/G ratio (~16) which was four times higher than that of the control and even higher than under saline stress, all of which is evidence of its ability to better survive desiccation when in the form of sessile cells attached to a support. A similar, although less marked, effect was observed for Rm1021 (B/G ratio ~5, Figure 5b).

### 2.5. Autoaggregation Assay

**Figure 6:**
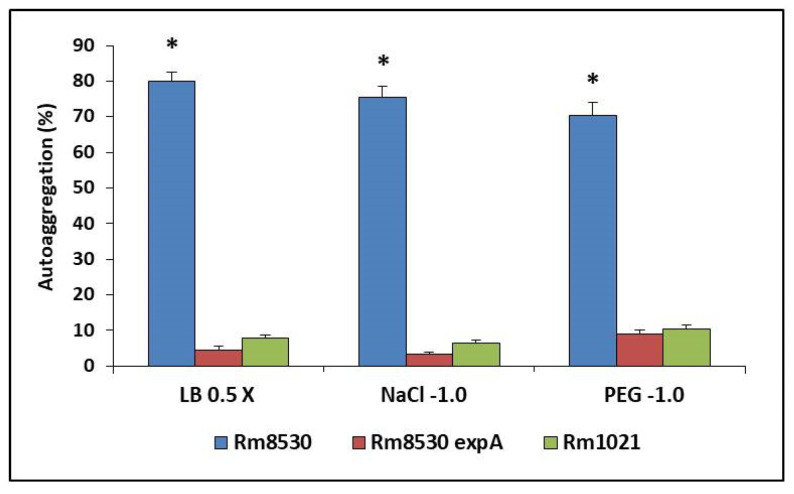
Quantitative autoaggregation assay of different *S. meliloti* strains under saline (NaCl) or matric (PEG) stress. The bars show the means of the autoaggregation percentages for Rm8530, Rm8530 *expA* and Rm1021, under NaCl- or PEG-induced water stress conditions (Ψ −1.0 MPa). The error bars represent the standard deviations from the means. Asterisks indicate statistically significant differences (*p* ≤ 0.05), according to Fisher’s LSD test.

Previous results have indicated that self-aggregation interactions in *S. meliloti* are mediated by EPS II [20]. The findings of the autoaggregation assay carried out as part of the present study clearly demonstrate that this effect is maintained under stress conditions created by a reduction in water potential (Ψ −0.5 MPa and −1.0 MPa). Since the effect at Ψ −0.5 MPa was similar to that at Ψ −1.0 MPa, only the results obtained under the most stressful condition are shown (Figure 6). EPS II-producing Rm8530 had autoaggregation values of around 80%, regardless of the specific stress conditions, whereas percentages were very low (<10%) under all conditions evaluated for non-producers (Figure 6).

## 3. Discussion

The findings from the water deficit assays presented here demonstrate that the cells of *S. meliloti* reference strain Rm8530 remain viable under this adverse condition. This observation could be linked to its ability to produce EPS II, a protective polysaccharide known to offer advantages in hostile environments against desiccation and other sources of biotic and abiotic stress [1]. The synthesis of symbiotically active EPS II requires a functional *expR* gene, which is intact in Rm8530. On the other hand, desiccation was accompanied by reduced viability in Rm1021 and Rm8530 *expA*. Wild-type Rm1021 produces detectable amounts of EPS I, but its *expR* gene carries an insertional mutation that results in a bigger-sized PCR product and prevents the synthesis of EPS II [4]. Mutant Rm8530 *expA*, for its part, is also defective in the biosynthesis of EPS II [20]. The inability to produce EPS II, therefore, could be behind the susceptibility to desiccation conditions of these last two strains. This falls in line with previous reports where we suggested that EPS II could provide resistance against toxic metals, probably by trapping the metal outside the cells and/or through biofilm formation [33].

Salt tolerance assays were carried out next by adding different NaCl concentrations to sandy supports on which the three strains were grown. Early studies indicate that *Rhizobium meliloti* SU-47 can tolerate concentrations of up to 0.2 M NaCl: higher concentrations than this either slow down growth or (at 1 M NaCl) directly stop it [34]. In the case of *S. meliloti*, certain NaCl concentrations can increase survival [35], a positive effect which could be related to the synthesis of trehalose and betain in osmotically stressed bacteria. Similarly, in our study Rm1021 was better able to survive at the lowest NaCl concentration tested, 155 mM, while exposure to higher concentrations (250 mM and 430 mM) decreased cell viability by five orders of magnitude towards the end of the assay. In contrast, Rm8530 and Rm8530 *expA* remained viable under all three concentrations. These results cannot be explained by the protective effect of EPS II, since only Rm8530 is capable of producing it. The response to salinity in Rm8530 strains, therefore, might be regulated by a global mechanism that is present in both of them but absent in Rm1021, perhaps with the involvement of the regulon affected by the transcriptional regulator ExpR. The effect of high salt concentration (300 mM) on *R. meliloti* EFB1 has been tested in previous experiments [32]. However, it should be kept in mind that these were artificially created salinity conditions. Natural soil is considered saline when its ion concentration interferes with the growth of agriculturally relevant species, and when it reaches an electrical conductivity of >4 dS m^−1^ (approximately 36 mM NaCl) measured in saturated soil at 25 °C [36].

On the other hand, the assessment of biofilm development under diminished water potential determined that it is dependent on the solute responsible for such effect and, once again, on the strains ability to synthesize EPS. Both saline (NaCl) and matric (PEG) stress affected growth, although the first appeared to stimulate it whereas the second prevented it from being normal. *S. meliloti* appears to have complex mechanisms to deal with saline stress, such as intracellular accumulation of osmolytes (organic solutes of low-molecular weight) or ions [29], modifications in the size and morphology of the cell [32], changes in transport proteins, and enzyme [37] and polysaccharide production [38]. Interestingly, the exposure of Rm1021 to osmotic stress was found to induce endoglycanase genes and to repress the *mucR* gene, which would lead to a reduction in EPS I synthesis and an activation of EPS II in that strain [39]. This is in agreement with our finding that growth, and consequently biofilm formation, increased for Rm1021 under saline stress, a condition under which a shift in the EPS synthesis pattern could be responsible for the phenotype observed. However, the increase in both parameters determined a similar B/G ratio between control and saline conditions, indicating that saline stress would not make a situation conditional on bacterial lifestyle in this strain. On the other hand, this bacterium seems less equipped to deal with desiccation stress, and among the processes affecting its survival under such conditions, the presence of EPS [35] and DNA-repairing systems [40], or lack thereof, seem to be crucial, as shown by the higher B/G ratio and the predominant bacterial sessile lifestyle. In short, our assays confirmed that EPS II may be one of the key elements for biofilm formation [41] and survival of *S. meliloti* under water reduction conditions. Water deficiency stress caused by a non-diffusible solute such as PEG, moreover, appears to be more determinant of sessile bacterial lifestyle than that induced by a diffusible solute such as NaCl. Although our assays were conducted in a reduced rich medium (LB 0.5X, the assessment of these parameters in minimal medium could provide another perspective on the role of biofilm development when nutrients are scarce (a situation that resembles more closely what happens in soils).

Taken together, the BFC results (Figure 5) and those corresponding to growth on plates (Table 1) support the notion that the EPS II-producing strain (Rm8530) opts for a biofilm lifestyle in the presence of salt, while the non EPS II-producing strains (Rm1021 and Rm8530 *expA*) preferentially keep a planktonic lifestyle, possibly using other mechanisms to cope with saline stress. Although Rm8530 grows less in salt, it forms more biofilm, a fact which is reflected in the higher mucus production detected on the plates. Nevertheless, those Rm8530 colonies that were mucoid due to the production of EPS II became dry in media with increased salt content, probably because the bacteria redirected their metabolic machinery towards another EPS synthesis profile and activated other mechanisms to cope with saline stress. A completely different process is likely to take place when bacteria are exposed to water potential reductions induced by non-diffusible solutes. Severe matric stress (−1.0 MPa) led bacterial cells to choose a sessile lifestyle, i.e., most of them became attached to a solid (abiotic) surface and managed to live as an associative biofilm community. This behavior was clearly observed in EPS II-producing Rm8530. Interestingly, even though Rm1021 is unable to produce EPS II, it was also capable of surviving by developing biofilm when matric stress was severe. Conversely, when matric stress was lower (−0.5 MPa), strains remained in their planktonic state even though growth was reduced. All in all, two scenarios might be triggered in bacteria when they sense stress at different intensities: (i) under slighter matric stress, bacteria may reduce growth while activating adaptive mechanisms to cope with the stress at low cell densities (i.e., DNA repair mechanisms), and (ii) under more severe desiccation conditions, they might quickly sense the threat and direct their entire metabolic efforts towards stopping planktonic growth and synthesizing the molecules (signals and effectors) required to establish a sessile lifestyle. In the case of Rm8530, this last process would probably be more successful thanks to its ability to produce EPS II. In the case of Rm1021, the biofilm matrix that enables survival under desiccation could be formed by other molecules. Alternatively, the ability to produce EPS II in this strain might be repressed in non-stressful conditions (perhaps through a MucR regulator) but become activated under desiccation stress (i.e., through *mucR* repression). These hypotheses need to be validated by adequate experimentation to further analyze biofilm.

Evidence related to autoaggregation in different *S. meliloti* strains suggests that while EPS I is not involved in the process under conditions of saline or matric stress, EPS II does play an important role, in addition to its involvement in structured biofilm formation. It has been previously shown that EPS II is precisely the EPS involved in the autoaggregative process and in the formation of structured biofilms in *S. meliloti* [20,41]. Thus, the results presented here are consistent with previous findings, although so far, the influence of EPS II under these specific experimental conditions has not been confirmed. Our results clearly show that this relevant property of microbial EPS II is maintained under water deficiency stress conditions (saline or matric), and thus offers bacteria protection against these adverse environmental situations. As previously explained, greater tolerance to environmental stress due to higher exopolysaccharide production could mean a greater ability to become grouped with other soil microbes that promote symbiosis [23], which would have an impact on microbial ecology as well as on alfalfa production.

The regulator ExpR controls the transcription of the *exp* genes involved in the production of symbiotically active EPS II. Therefore, the presence of an IS element in the *expr* gene of *S. meliloti* Rm1021 affects EPS II production. In any case, this insertional mutation could have a pleiotropic effect. ExpR behaves as a global transcriptional regulator; more than 500 genes have been identified as differentially expressed by ExpR and it is the major regulator of AHL-controlled gene expression in *S. meliloti* [42]. Indeed, the ExpR/Sin QS system has been shown to play a central role in *S. meliloti* gene expression [8,9], including the control of motility gene expression as a result of the VisN/VisR/Rem relay [43]. Nevertheless, the responses and phenotypes associated with these global regulators could be related to each other, since the production of EPS II by cells harboring a functional ExpR/Sin system enables certain types of motility phenotypes that are independent of flagellar activity [44]. All of this offers evidence on the complexity of the regulation in which ExpR is involved. Furthermore, we observed that a direct connection cannot be established between survival rate under saline stress and EPS II production. The phenotypes observed here, then, might not respond exclusively to a change in EPS II production, at least under these experimental conditions. However, the production of this polysaccharide certainly appears to be a relevant factor for the survival of *S. meliloti* under certain adverse environmental conditions.

Finally, the results presented here indicate that the tendency to establish cell–cell interactions in *S. meliloti* (a critical aspect for the formation of biofilms) could be already present in the cells living planktonically and become manifested under stress conditions such as those in the aggregation experiments. Bacteria in a planktonic or sessile state which are capable of synthesizing EPS are better equipped to withstand exposure to environmental stress coming from reduced water potential. Despite the fact that the transition from one lifestyle to another depends on the modification of gene expression patterns and bacterial physiology [45,46], the synthesis of EPS II seems to be a common crucial characteristic for the occurrence of both biofilm formation and autoaggregation.

## 4. Materials and Methods

### 4.1. Bacterial Strains and Growth Conditions

The strains used in this study are listed in Table 2. Cells from agar plates were inoculated into a liquid culture in a shake flask containing TY broth [47]. The antibiotics used were streptomycin (500 µg/mL) and gentamycin (40 µg/mL). The cultures were incubated at 30 °C on a rotary shaker (Model SI4-2 Shel Lab, 12 mm orbit, Sheldon Manufacturing Inc., Cornelius, OR, USA) at 200 rpm, as described previously [20]. The CF-bright phenotype was observed on agar plates containing Luria-Bertani (LB) supplemented with 200 μg/mL of CF White M2R (Sigma Aldrich, St. Louis, MO, USA). CR-staining was performed on MGM agar plates supplemented with 125 μg/mL CR.

### 4.2. Bacterial Cell Viability Assays under Specific Stress Conditions

In order to assess potential survival, bacterial strains Rm1021, Rm8530, and Rm8530 *expA* were inoculated into sand where different stress conditions were generated. The number of viable cells per gram of sand was recorded after incubation at different times (0, 7, 20, 30, and 40 days).

#### 4.2.1. Water Stress

Two grams of sand filtered through a 0.4 mm sieve were placed in test tubes and then moistened until field capacity was reached with a saline solution (NaCl 0.9% *w*/*v*) containing *S. meliloti* strains, in order to establish 1 × 10^6^ CFU per gram of sand. The tubes were plugged with cotton plugs and incubated for the indicated times at 30 °C. Tubes sealed with parafilm to prevent water loss were included for each strain as controls without dehydration. Then, moisture percentage was evaluated by weighing. Sand from each control and treated tube was removed, weighed, and dehydrated in a stove at 60 °C until constant weight was reached. Finally, moisture percentages were obtained following this equation: %H: (Psh + T) − (Pss + T)/(Pss + T) − T; where Psh = weight of damp sand, Pss = weight of dry sand and T = tare. Moisture (H%) content measurements were made both to check whether the experimental design was indeed effective, and to assess how the stressful condition was established across time (Table 3).

To determine the total number of viable bacteria, 2 mL of sterile saline solution containing 0.01% Tween 80 were added to each tube. After 15 min of vortex agitation, appropriate 10-fold dilutions were made, and each dilution was plated out on Petri dishes containing solid LB medium. After the incubation time required, the CFU g^−1^ of sand was recorded. Each test was repeated five times with three replicates each.

#### 4.2.2. Saline Stress

Two grams of sterile sand were placed in test tubes and then moistened to field capacity with NaCl salt solutions thus prepared: 430 mM (2.5% *w*/*v*), 250 mM (1.5% *w*/*v*), and 155 mM (0.9% *w*/*v*, control tubes). Each tube was inoculated with each *S. meliloti* strain to establish 1 × 10^6^ CFU per gram of sand. The tubes were sealed with parafilm and incubated for different periods of time at 30 °C. Bacterial count was performed as described previously. Each test was repeated five times with three replicates each.

#### 4.2.3. Strain Growth and Colony Morphology on Saline Stress Plates

Strain growth and colony appearance (dry or mucoid) under saline stress were evaluated on agar plates (1.5% *w*/*v* agar) reduced LB medium (0.25X, −0.14 MPa water potential) to which different amounts of NaCl were added to obtain media with reduced water potential (−0.65, −1.15, −1.65, and −2.65 MPa). Assays under matric stress (PEG 8000 addition) could not be carried out due to the impossibility of dissolving the PEG 8000 solute in LB media containing agar.

### 4.3. BFC Test

BFC was determined by quantitative analysis using 96-well ELISA plates, as described by O’Toole and Kolter [50]. Pre-cultures were made in two mL of TY medium and incubated under agitation for 48 h at 30 °C. Bacterial cultures were diluted with fresh medium to reach an absorbance at 620 nm (OD620) of 0.01. Then, 150 µL of each suspension were added into each well and incubated for 24 h at 30 °C. Bacterial growths was quantified by measuring the OD of the planktonic cells in each well at 620 nm with MicroELISA (Series 700 Microplate Reader, Cambridge Technology, Watertown, MA, USA.). Planktonic cells were removed, each well was washed three times with saline solution, and the cells attached to the support were stained with 180 µL of Crystal Violet (0.1% *w*/*v*) for 15 min. The wells were rinsed several times with sterile distilled water and biofilm formation was quantified by adding 150 µL of 95% ethanol and by measuring the OD of the solubilized crystal violet at 570 nm (OD570). Simultaneously, controls without bacteria were carried out. Relative BFC was calculated as OD570/OD620 ratio.

#### BFC Test under Stress Conditions

Biofilm formation by different *S. meliloti* strains was evaluated in diluted LB medium (0.5X), which generates an osmotic potential of −0.25 MPa. Different amounts of NaCl or PEG 8000 were added to this medium to create different conditions of water potential reduction (saline or matric stress, respectively), with values of −0.5 and −1.0 MPa for each solute.

### 4.4. Autoaggregation Assay

Autoaggregation studies were performed as previously described [20]. Briefly, each *S. meliloti* strain was grown in 2 mL of LB medium for 24 h at 30 °C. Then, 100 µL of the bacteria were subcultured in 100 mL of LB medium, and incubated for 48 h in the previously described conditions. Next, suspensions were transferred to glass tubes and kept at 4 °C for 24 h. A 0.2 mL aliquot from the upper portion of the suspension was carefully transferred to a microtiter plate, and OD600 was measured as ODfinal (ODf). A control tube was vortexed and OD600 was determined as ODinitial (ODi). Autoaggregation percentage was calculated as 100 [1-ODf/ODi].

### 4.5. Statistical Analysis

The experiments were carried out using randomized designs, with the values representing the averages of at least three replicates, depending on the experiments. The data were analyzed using ANOVA, and multiple variables were compared through Fisher’s LSD test. The level of significance was set at *p* = 0.05. All the statistical analyses were made on Infostat 1.0 (InfostatGroup, Universidad Nacional de Córdoba, Córdoba, Argentina).

## Figures and Tables

**Table 1:** Growth and colony morphology under saline stress conditions. *S. meliloti* strains were streaked on plates of LB (0.25X) medium with different water potentials (Ψ). Growth was assessed semi-quantitatively as: Considerable Growth (CG), Little Growth (LG) and Scarce/No Growth (SG/NG). The phenotype of colony morphology was defined as mucoid (M) or dry (D). * The numbers between parentheses correspond to the reduction in water potential caused by the addition of NaCl in excess to that already present in the medium.

		Strains		
LB Medium (0.25X)		Rm1021		Rm8530
Addition of NaCl (g L^−1^)	Ψ (MPa) *	wt	wt	*expA*
1.25	−0.15	CG (D)	CG (M)	CG (D)
6.4	−0.65 (−0.5)	CG (D)	CG (M)	CG (D)
12.8	−1.15 (−1.0)	CG (D)	CG (D)	CG (D)
19.2	−1.65 (−1.5)	LG (D)	LG (D)	LG (D)
32.0	−2.65 (−2.5)	SG (D)	SG (D)	NG

**Table 2:** Bacterial strains used in this study.

*S. meliloti* Strain	Relevant Properties	EPS I/EPS II Phenotype	Reference
Rm1021	SU47 *str21 expR102*::IS*Rm*2011-1 (*expR^-^*)	EPS I	[48]
Rm8530	SU47 *str21 expR101* (*expR^+^*)	EPS I/EPS II	[49]
Rm8530 *expA*	*expA3*::Tn5-233 (*expR^+^*)	EPS I	[20]

**Table 3:** Moisture percentages throughout the viability experiment. Time-dependent humidity percentage (H%) is indicated for each strain both in tubes subjected to stress (ST) and in control tubes (CT) without dehydration.

	Strains					
	Rm1021		Rm8530			Rm8530 *expA*
Time (Days)	CT (H%)	ST (H%)	CT (H%)	ST (H%)	CT (H%)	ST (H%)
0	22.1	22.1	23.1	23.1	19.7	19.7
7	15.0	8.2	15.3	8.6	17.7	8.6
20	15.0	4.0	15.5	4.0	16.0	6.0
30	15.0	3.0	16.0	3.5	15.0	5.0
40	16.0	0.4	16.5	0.3	16.0	0.4
